# Integrating anatomy and function for zebrafish circuit analysis

**DOI:** 10.3389/fncir.2013.00074

**Published:** 2013-04-23

**Authors:** Aristides B. Arrenberg, Wolfgang Driever

**Affiliations:** Developmental Biology, Institute of Biology I, Faculty of Biology, BIOSS - Centre for Biological Signalling Studies, Albert-Ludwigs-University FreiburgFreiburg, Germany

**Keywords:** zebrafish model system, optogenetics, neural circuits, calcium imaging, brain anatomy, neuronal types

## Abstract

Due to its transparency, virtually every brain structure of the larval zebrafish is accessible to light-based interrogation of circuit function. Advanced stimulation techniques allow the activation of optogenetic actuators at different resolution levels, and genetically encoded calcium indicators report the activity of a large proportion of neurons in the CNS. Large datasets result and need to be analyzed to identify cells that have specific properties—e.g., activity correlation to sensory stimulation or behavior. Advances in three-dimensional (3D) functional mapping in zebrafish are promising; however, the mere coordinates of implicated neurons are not sufficient. To comprehensively understand circuit function, these functional maps need to be placed into the proper context of morphological features and projection patterns, neurotransmitter phenotypes, and key anatomical landmarks. We discuss the prospect of merging functional and anatomical data in an integrated atlas from the perspective of our work on long-range dopaminergic neuromodulation and the oculomotor system. We propose that such a resource would help researchers to surpass current hurdles in circuit analysis to achieve an integrated understanding of anatomy and function.

## The zebrafish as a genetic model for neural system and behavioral analysis

About 30 years after George Streisinger introduced zebrafish as a model system (Streisinger et al., 1981), zebrafish continues to be an attractive animal model. Initially, investigators were fascinated by this small vertebrate because the external development of zebrafish embryos and the favorable husbandry were advantageous for developmental and genetic studies. Over the past two decades, researchers started to increasingly use zebrafish as a model for understanding how neuronal circuits generate behavior, a fundamental goal in neuroscience. Mutagenesis screens revealed genes that are important for proper brain development or required for sensory processing (Brockerhoff et al., 1995; Neuhauss et al., 1999; Muto et al., 2005) or locomotion (Granato et al., 1996). Many of these mutants only affect the sensory or motor periphery and provided little insight into central brain processing. Since gene functions often affect multiple neuronal groups when mutated, mutational dissection of individual contributions to defined circuits is impeded (as discussed in Gahtan and Baier, 2004; McLean and Fetcho, 2008). Therefore, new tools were needed that enable to probe brain function locally after normal development has taken place. Optogenetic tools perfectly fit this purpose and are providing another boost to this already much appreciated animal model.

## Optogenetic actuators

The discovery of light-activated channels and transporters in single-cell organisms (Schobert and Lanyi, 1982; Kalaidzidis et al., 1998; Nagel, 2002, 2003) and their subsequent incorporation into neurons (Zhang et al., 2007; Fenno et al., 2011) established a highly-versatile optogenetic toolbox for conditionally and reversibly manipulating brain activity in zebrafish (Szobota et al., 2007; Douglass et al., 2008; Arrenberg et al., 2009; Wyart et al., 2009; Zhu et al., 2009; reviewed in Baier and Scott, 2009; McLean and Fetcho, 2011). These actuators enable unprecedented spatial and temporal resolution of activity perturbation. For example, Warp et al. (2012) investigated the development of the spinal central pattern generator using these tools. Initially, the cells of the future central pattern generator are sporadically and seemingly randomly active. They then develop into ipsilaterally correlated clusters and eventually form a single ipsilateral network on each side producing the alternating left-right excitation needed during swimming. The authors used brief local halorhodopsin stimulations and concurrent calcium imaging to reveal the development of coupling between the targeted cell and neighboring cells during the period from 18 to 21 h post fertilization. Using chronic halorhodopsin stimulations, the authors furthermore showed that the development of coupled, synchronous activity is dependent on activity. This study exemplifies the power of targeted optogenetic manipulations for answering questions regarding the activity-dependent development of networks.

**Figure 1:**
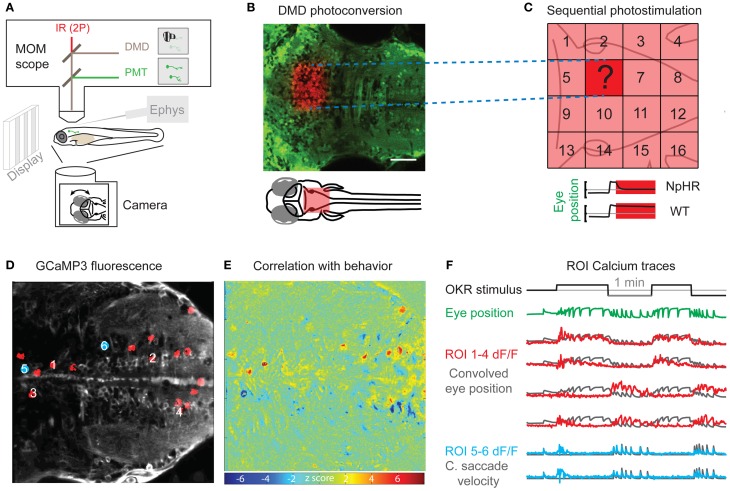
**Perturbation and Measurement of brain activity via optogenetics. (A)** Scheme of a moveable objective microscope (MOM) that allows two photon (2P) calcium imaging (PMT, photomultiplier tubes) and photostimulation via a digital micro mirror device (DMD). At the same time, the animal can be visually stimulated and eye or tail movements are recorded with a camera. **(B)** A rectangular region of interest was photoconverted with the DMD device in an *Et(E1b:Gal4)s1101t; Tg(UAS:Kaede)s1999t* transgenic animal. **(C)** Schematic for the systematic mapping of circuit function by measuring behavioral performance (e.g., eye position stability) during sequential photostimulation (16 regions shown here) in animals transgenic for optogenetic actuators (e.g., halorhodopsin, NpHR). The question mark illustrates an individual tested region as shown in **(B)**. **(D)** Averaged frame of a calcium imaging time series during visual optokinetic response (OKR) stimulation in an *Et(E1b:Gal4)s1101t; Tg(UAS:GCaMP3)* transgenic 5-day-old animal. Red regions of interest (ROIs) correspond to highly correlated pixels in **(E)**. Blue regions of interest are correlated with quick eye movements. **(E)** A heat map of Z-scores identifies pixels correlated with average eye position. The algorithm is based on Miri et al. (2011b) and provides an unbiased, quick way of correlating pixel time series with any time-varying regressor of interest. The Z-score heat map for correlation with saccadic eye movements [blue positions in **(D)**] is not shown. **(F)** The black line indicates the velocity of the visual stimulus (vertical stripes) and the green line shows the average eye position. The calcium fluorescence ΔF/F signal of the ROIs 1–4 in **(D)** (red) is plotted over eye position regressor traces (black). ROIs 1 and 2 have a positive correlation with the eye position regressor and ROIs 3 and 4 are anti-correlated with the eye position regressor. The lower two calcium signal traces (blue) correspond to the blue positions in **(D)** and are correlated with the occurrence of quick eye movements (saccades). The black traces show the high-pass filtered eye velocity regressor. Note that all six black regressors were convolved to account for the slow calcium indicator kinetics. Scale bar: 50 μm.

Apart from genetic targeting of selected cell types, the specificity obtained with optogenetic expression can be improved by limiting the illumination volume in the brain. Several optical stimulation techniques are available and can be chosen based on the required spatial resolution. Fiber-optic stimulations (Arrenberg et al., 2009) provide a method for low resolution localizing circuit function. Digital micromirror devices (DMDs)—a technology used in video projectors—are an attractive option, because they allow the simultaneous stimulation in any combination of pixels of the photostimulation mask and provide focal stimulation when mounted on a microscope (Wyart et al., 2009; Arrenberg et al., 2010; Blumhagen et al., 2011; Zhu et al., 2012). Such a DMD device can be used to map functions in the focal plane of interest (Figure 1). However, a one-photon optical focus generates considerable light excitation in out-of-focus planes, especially when the effective numerical aperture of the objective is small. Ideally, the expression can be genetically restricted to the cells of interest, thus evading problems regarding off-target stimulation. While the local activation of small groups of genetically defined neurons will be sufficient in many experiments, other experiments will require the stimulation of single cells. To accomplish single cell stimulation, more sophisticated photostimulation techniques are likely needed, such as holographic stimulation (Lutz et al., 2008), two-photon stimulation using temporally focused beams (Andrasfalvy et al., 2010; Papagiakoumou et al., 2010; Oron et al., 2012), or quick scanning of the membrane surface of the cell (Rickgauer and Tank, 2009; Zhu et al., 2009).

## Holistic calcium imaging

While optogenetic activation and inhibition experiments directly test the causal link between circuit modules and behavior, these experiments typically require sequential point-by-point testing of brain tissues. In contrast, activity probes such as calcium indicators are complementary tools and enable simultaneous read-out of brain activity from a large number of neurons, thereby accumulating correlative evidence. To optically record neural activity, zebrafish scientists have mainly relied on local injections of synthetic calcium indicator dyes in the past, which - among others - advanced our knowledge about reticulospinal cells (O'Malley et al., 1996; Liu and Fetcho, 1999; Gahtan et al., 2002; Orger et al., 2008), visual circuitry (Niell and Smith, 2005; Sumbre et al., 2008), olfaction (Friedrich and Korsching, 1997), and motor circuitry (Fetcho and O'Malley, 1995). With synthetic organic calcium indicators, only the fraction of cells that took up the injected calcium indicator can be imaged. In contrast, genetically encoded calcium indicators (Grienberger and Konnerth, 2012; Knöpfel, 2012) enable genetic targeting of neurons. When combined with (almost) pan-neuronal promoters, homogeneous expression of genetically encoded calcium indicators throughout the CNS can be achieved and used to study function of whole brain areas in a non-biased way (Niell and Smith, 2005; Aizenberg and Schuman, 2011; Tao et al., 2011; Ahrens et al., 2012). The calcium signal of hundreds of cells can be imaged concurrently and automated algorithms can be applied to calculate the correlation of each pixel in the calcium imaging fluorescence time series with any aspect of the time-varying behaviors or sensory stimuli. Efficient algorithms have been developed (Miri et al., 2011b) and allow for quick identification of correlated pixels (and then neurons) by regressing pixel time series with any regressor of choice (e.g., eye position in Figure 1). Since a large number of cells can be imaged concurrently, the small size of the larval brain [roughly 500 × 500 × 1000 μm, 100,000 neurons (Hill et al., 2003)] opens the prospect to sequentially record the activity of all cells in a single animal (here, we refer to this whole brain approach as “holistic”). Using light sheet microscopy with a sCMOS camera, Ahrens and Keller (2013) achieved 1.3 s temporal resolution for recording calcium imaging stacks covering about 90 percent of the 5-day-old larval brain volume at single cell resolution. This work demonstrated that whole brain functional imaging at cellular resolution is well feasible in zebrafish larva.

Whole brain calcium imaging at cellular resolution requires sensitive genetically encoded calcium indicators and homogeneous expression in order to allow activity recording in all neurons of the animal. In a pioneering study, Ahrens et al. (2012) reported on brain-wide activity at cellular resolution during a motor learning paradigm. The authors identified activity that was correlated with a visual stimulus, fictive swimming behavior, or with altered motor performance. This study, which elegantly illustrated the power of holistic calcium recording, also revealed some of its limitations. Due to the relatively low sensitivity of the genetically encoded calcium indicator used, GCaMP2 (Tallini et al., 2006), much of the brain activity was likely missed, and the authors accordingly report that activity could only be detected in about 1% of the cells during the experiments. More recent calcium indicators, like GCaMP5G (Akerboom et al., 2012) already allow to observe activity in a much larger fraction of neurons. In the study by Akerboom et al., GCaMP5G, GCaMP3, and the synthetic indicator OGB-1-AM were compared side by side in a test of visual responsiveness in mouse cortex. While GCaMP5G was twice as good as GCaMP3 (20 vs. 10% of the expressing cells were visually responsive), it still lagged the performance of the synthetic dye OGB-1-AM, for which almost 40% of the cells responded. A low calcium indicator sensitivity also biases toward detection of large calcium events, while low firing rates of responsive cells are likely missed. In the above mentioned light sheet microscopy study, Ahrens and Keller (2013) already used a recent genetically encoded calcium indicator, GCaMP5G, for whole brain imaging, which should allow improved activity detection compared to the previous study with GCaMP2 (Ahrens et al., 2012). However, the fraction of responsive cells was not reported. Genetically encoded activity probes with improved sensitivities and linear dynamic ranges are in demand and will likely be developed in the near future (Akemann et al., 2012; Chen et al., 2012; Muto et al., 2013). In addition, suitable promoters for the homogeneous pan-neuronal expression need to be identified to avoid differences in expression levels (e.g., the HuC/elavl3 promoter induces only very low levels of expression in the diencephalon at 5 dpf). Furthermore, the cytosolic expression of genetically encoded calcium indicators (and their exclusion from the nucleus) results in strong neurite signals, and complicates the analysis of cellular signals. Alternative expression strategies, e.g. using nuclear localization sequences, could facilitate the differentiation between somatic and neurite signals.

While there is still room for improvement of technology, zebrafish larva now provide the opportunity to look at the brain's response in a holistic fashion at cellular resolution, which has not been possible in any vertebrate model organism before. Correlations of activity in distant brain regions could be revealed, that would be very hard to show by electrophysiology or in bigger animals. For example, regarding the dopaminergic system, the long-range neuromodulatory A11 dopaminergic system could be imaged to study the integration of dopaminergic modulation from the telencephalon to the spinal cord (Tay et al., 2011).

## Behavior during holistic imaging

While the sensitivity of calcium indicators is limiting on the activity detection side, the behavioral paradigms that can be elicited in an immobilized preparation are crucially limiting the extent to which functional analyses can be performed during whole brain calcium imaging. For circuit neuroscience, the usefulness of zebrafish holistic imaging scales with the number and quality of applicable behavioral or sensory stimulation paradigms. Good candidates for the rapid generation of functional data are neurons that process sensory information, since their activity likely does not rely on proper behavioral performance, and neurons mediating robust reflex behaviors, e.g., the escape response, the optokinetic response, and the optomotor response. Other behaviors might require appropriate sensory feedback during unrestrained swimming, or lengthy experimental protocols. Such behaviors are therefore more difficult to analyze in immobilized fish under a microscope, e.g., prey capture (Bianco et al., 2011), the diving response during dark photokinesis (Fernandes et al., 2012), or learning paradigms (Aizenberg and Schuman, 2011; Wolman et al., 2011; Valente et al., 2012). Improvements in animal mounting, monitoring, and feedback methods, including closed-loop fictive swimming paradigms (Ahrens et al., 2012), will likely facilitate the development of behavioral paradigms in immobilized preparations.

## Anatomical annotation in larval zebrafish

Calcium imaging in larval zebrafish enables the generation of three-dimensional (3D) maps of whole brain activity, similar in anatomical scale to data obtained from functional magnetic resonance imaging (fMRI) in larger animals. However, whole brain calcium imaging in zebrafish has the benefits of direct activity measurement and high (sub-cellular) spatial and temporal resolution. To reveal the cellular identities of the imaged brain volumes, a correct anatomical framework needs to be available. Anatomical atlases and gene expression databases already exist for zebrafish and are listed in Table 1, including links and references for the resources mentioned in the following text. Some resources include anatomical annotations in the absence of expression markers (ZFIN anatomy resources, PSU Zebrafish Atlas). Furthermore, detailed anatomical ontologies are curated (ZFIN anatomical ontologies), but their application to larval stages is handicapped by the fact that the ontologies rely on morphologically identifiable structures, which are scarce in the larval zebrafish brain. Therefore, the larval brain is somewhat “under-annotated,” which will only improve once gene expression data are integrated with morphology data to define larval brain structures. Other atlas initiatives include expression analysis [ZFIN database; Zebrafish Brain Atlas; hardcopy atlas by Mueller and Wullimann (2005)]. However, the precise anatomical annotation is a current hurdle in zebrafish circuit neuroscience.

**Table 1:** **Selected anatomical resources for the larval and adult zebrafish brain**.

**Name of resource**	**Internet address**	**Developmental stages**	**Available information**	**Authors**
ZFIN	http://zfin.org/action/anatomy/anatomy-search; http://www.berkeleybop.org/ontologies/zfa.obo	Any	Detailed anatomical ontologies	Zebrafish Anatomical Dictionary Workgroup http://zfin.org/zf_info/anatomy/dict/mem.html
	http://zfin.org/cgi-bin/webdriver?MIval=aa-xpatselect.apg	Any	Expression database linking to gene expression publications	Bradford et al., 2011
	http://zfin.org/zf_info/anatomy/dict/sum.html	1, 2, 3, 5 dpf	Annotated anatomical sections	Workgroup see: http://zfin.org/zf_info/anatomy.html
Zebrafish Atlas	http://zfatlas.psu.edu	2, 3, 4, 5, 6, 7–13, 14–20, 21–29 dpf, and up to 12 months	Annotated anatomical sections; coronal, sagittal, and transversal	Cheng and Copper, 2012 (Jake Gittlen Cancer Research Foundation)
Zebrafish Brain Atlas	http://www.zebrafishbrain.org/	Several	Chapters on different brain regions and systems, making use of transgenic lines	Wilson et al., 2012
Atlas of Early Zebrafish Brain Development	Hardcopy atlas	2, 3, 4, 5 dpf	Neuroanatomical expression atlas	Mueller and Wullimann, 2005
Neuroanatomy of the Zebrafish Brain	Hardcopy atlas	Adult	Neuroanatomical structures	Wullimann et al., 1996
Virtual Brain Explorer for Zebrafish (ViBE-Z)	http://vibez.informatik.uni-freiburg.de	2, 3, 4 dpf	Tool to register 3D datasets to reference brain, small database with aligned gene expression patterns	Ronneberger et al., 2012

Work in rodents allows stereotactic targeting of identified brain nuclei [e.g., hard copy-atlas from Franklin and Paxinos (2012)]. While the transparency of larval zebrafish should facilitate identification of stereotactic landmarks, the small size of larval zebrafish, the non-rigid consistency of the larva, and potential inter-larval shape-differences complicate the development of stereotactic methods. If larval zebrafish had very stereotypic, rigid shapes, three landmarks should suffice to calculate the rotation and translation of the animal and its neural structures with respect to a reference animal. However, such straightforward registration approaches are not precise enough to reliably identify small cell groups. For example, Ahrens et al. (2012) report on a precision of only 25 μm when registering microscopical optical slices to a reference brain using rigid translation.

Transgenic zebrafish lines expressing fluorescent proteins under the control of specific promoters are frequently used to identify specific neuronal groups or provide additional landmarks. However, only a small number of markers can be used in parallel in any given experiment. In addition, the unavailability of suitable promoters frequently necessitates identification by other means, e.g., based on activity or projection patterns. Therefore, a serial strategy would be beneficial, where gene expression and functional information from different locations in the brain, different times, and different laboratories can be placed and registered to a reference, allowing the community to build a multi-dimensional brain map. While many functional neuronal entities form tight spatial clusters—e.g., cranial nuclei (Higashijima et al., 2000) and dopaminergic cell groups (Filippi et al., 2010)—other neuronal entities might be less clustered and intermingle with cells having non-related functions—e.g., cells of the neural integrator for horizontal eye movements do not appear to be tightly clustered (Miri et al., 2011a). For larval zebrafish, therefore, the combination of activity imaging data from several specimens will not suffice to identify anatomically distinct nuclei, but generate likelihood clouds positioning circuit activity components in an anatomical framework. Linking such likelihood cloud data to molecular markers from gene expression analysis of transgenic lines may then enable to identify the neuronal populations involved, and also to relate them to late juvenile and adult brain structures.

## Virtual three-dimensional anatomical frameworks

For several animal models, 3D databases have been developed to begin to integrate morphological, gene expression, and functional data. In *C. elegans*, the defined cell lineage provides a framework for such databases. For *Drosophila*, cellular resolution has been achieved for registration of different brains (BrainAligner, Peng et al., 2011), while for mouse and human such databases operate at a more coarse anatomical level (Allen Brain Atlas, http://www.brain-map.org and MGI Mouse Genome Informatics Jackson, http://www.informatics.jax.org, Finger et al., 2011). However, for zebrafish, none of the databases listed in the last paragraph offers a framework in which scientists could use their own expression data and align it to already existing data in a digital atlas.

**Figure 2:**
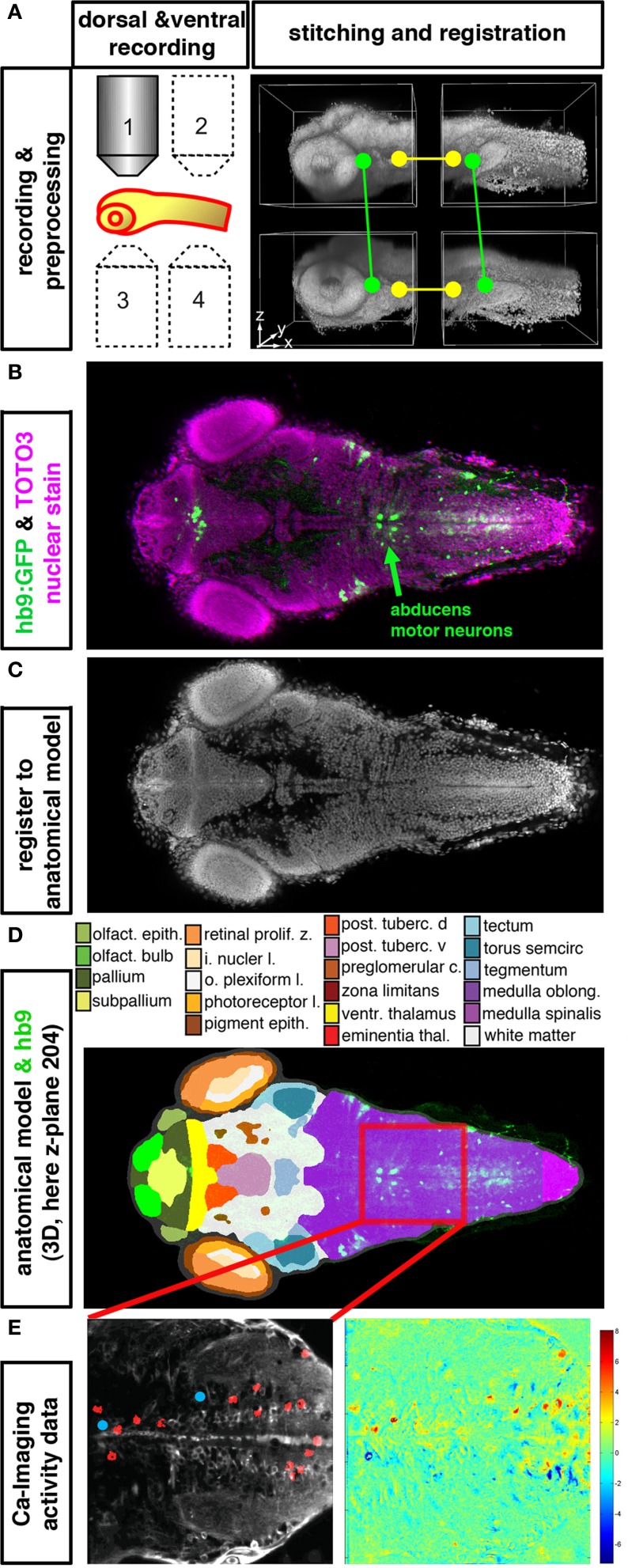
**Integration of anatomical, gene expression, and functional information in 3D. (A–E)** High resolution datasets generated using an anatomical reference stain for each developmental stage may serve to establish an anatomical reference to register different data types into an anatomical and functional atlas of the nervous system. The Virtual Brain Explorer for Zebrafish (ViBE-Z) provides a framework for generation of high resolution 3D image stacks and for registration into an anatomical model (Ronneberger et al., 2012). **(A)** For single cell resolution documentation of the whole zebrafish brain, image stacks of rostral and caudal parts of the brain are recorded from ventral and dorsal sides using standard single photon confocal microscopes. Fluorescent stain of all cell nuclei is used to obtain anatomical information, and also to correct for loss of signal in deep tissues, assuming that all nuclei contain the same amount of DNA and have similar stain intensities. ViBE-Z stitches the individual stacks into one high resolution 3D data volume and performs correction of optical attenuation (light absorption and scattering in tissue). **(B)** Example of a ViBE-Z processed dataset of a 3-day-old larva showing GFP expression from *Tg(hb9:GFP)* (Flanagan-Steet et al., 2005) and TOTO3 fluorescent nuclear stain. **(C)** The fluorescent stain of cell nuclei is used by ViBE-Z to extract landmark information and register the experimental datasets in 3D to a reference larva. **(D)** The anatomical model of the reference embryo may now be combined with the experimental data set. Here, a dorsal view at focal plane z_204 of the reference larva is shown. The experimental *Tg(hb9:GFP)* expression provides neuronal information even in the medulla oblongata hindbrain region, which has only few anatomical annotations in reference databases (www.ZFIN.org). **(E)** In the future, as soon as anatomical references may be recorded also in live larva, the anatomical and gene expression information in ViBE-Z may be used to identify neurons which have defined recorded activity patterns. In this case, neurons with activity patterns correlating with oculomotor activity have been detected (see Figure 1), and ViBE-Z may be used to determine whether some of these active neurons may correspond to abducens motor neurons, which are labeled in the *Tg(hb9:GFP)* transgenic line.

To address this deficit, our group recently developed the Virtual Brain Explorer for Zebrafish (ViBE-Z), a microscopy and computational framework to align expression pattern data to a reference brain (Ronneberger et al., 2012; “http://vibez.informatik.uni-freiburg.de”). ViBE-Z detects characteristic landmarks in the fish embryo and early larva based on fluorescent stain of all cell nuclei. Landmarks and subsequent fine elastic registration are used to register experimental data to a reference brain, thereby accounting for inter-fish differences resulting from variations in brain morphology or staining artifacts. The atlas has a precision of about 2 μm, so that expression comparisons can be performed at near-cellular resolution (Figure 2). ViBE-Z facilitates the characterization of neuronal identities and subtypes, and also helps to postulate potential regulatory mechanisms based on genetic intersectional strategies. Although ViBE-Z can serve as a tool to define likelihood clouds for expression domains, it cannot predict expression in individual neurons across different brains since the positions of individual defined neurons are variable. It also does not—as of yet—provide a means to integrate *in vivo* functional data, and thus contribute to understand the circuits that certain brain volumes are involved in. Furthermore, Vibe-Z only includes the positions of somata and does not take into account the position and targets of neuronal processes.

## Integration of diverse data types in a virtual framework

We propose that a framework like the ViBE-Z atlas could be used to include maps of projection patterns, neurotransmitter identities, as well as maps of functional data. The mapping of reticulospinal connections (Kimmel et al., 1982) has been a good example of how the establishment of projection information can serve as a seeding point for scientific discovery. The reticulospinal cells are relatively few in number and therefore provide a bottleneck in relaying swim commands to the spinal cord. Identified sets of reticulospinal neurons control escape responses (O'Malley et al., 1996; Liu and Fetcho, 1999), aspects of prey capture (Gahtan et al., 2005), and swim turns (Orger et al., 2008). Regarding other “projectomes” (Kasthuri and Lichtman, 2007), for zebrafish the complete projection patterns of dopaminergic and noradrenergic neurons have been established (Tay et al., 2011), revealing that generation of such data is possible for long-range projection systems in zebrafish. The projectome of Otp-dependent diencephalic dopaminergic cell clusters of the A11 type revealed both ascending projections to the telencephalon and long-range projections into the hindbrain and spinal cord, providing a new framework to study A11 dopamine neurons in vertebrates. In the cases of reticulospinal cells and dopaminergic cells, projection patterns have been established based on projection target and neurotransmitter identity, respectively. In the future, the application of serial block face electron microscopy and other methods like trans-synaptic viral tracing or the use of photoactivatable/-convertible fluorophores offer the potential to contribute to the generation of rich projectome information (Denk et al., 2012).

In order to fully exploit the potential of whole brain calcium imaging we need to know the identities of the recorded cells. A particularly meaningful marker in many circuits is the type of neurotransmitter the cells are using. For example, in the case of the neural integrator for horizontal eye movements, the precise connectivity of the integrator network is not known, although electrophysiological data (Aksay et al., 2000, 2003) suggests that glutamatergic cells of the integrator are auto-excitatory to the ipsilateral side, whereas inhibitory integrator cells are likely GABAergic and inhibit the contralateral side. Recent work on the arrangement of hindbrain cell types (Kinkhabwala et al., 2011) revealed that hindbrain circuitry is made up of stripes of cells with specific wiring properties. The neurotransmitter type and medio-lateral positioning in the hindbrain together thus affect whether the cells have ascending, descending, ipsilateral or contralateral projections or a combination of different projection types. Therefore, the knowledge of neurotransmitter type will - together with the precisely annotated position of the cells - greatly facilitate the understanding of the functions of oculomotor system cells' calcium signals.

The generation of 3D activity maps will help to identify neuronal groups correlated with behavioral functions. To test the function of these neurons causally, perturbation maps in which brain volumes are systematically activated or silenced during behavior (see Figure 1C), could be generated at different levels of resolution. In bigger animals, electrical stimulation has been used to identify topographic maps of behavioral function. For example, in the superior colliculus of monkeys, cats and goldfish, saccades can be elicited by electrical stimulation and the location of stimulation affects the magnitude and direction of the saccades in a topographic manner (Robinson, 1972; Guitton et al., 1980; Angeles Luque et al., 2005). Similar perturbation maps could be generated for larval zebrafish making use of optogenetics and high-resolution photostimulation. Inclusion of projectomes and functional data in a potential digital atlas of the future will facilitate pathway mapping and advance our understanding of brain functions.

To be most useful, a digital reference atlas should allow researchers to add their own functional and anatomical data to the atlas. In the current ViBE-Z implementation, this is facilitated by using a simple fluorescent stain of cell nuclei as anatomical reference stain. However, in the future one would also like to implement data from live brain recordings. Therefore, a widely available reference fish line would be useful that may fluorescently label a sufficient number of structures to enable landmark detection and fine elastic registration. While some promoters might be broadly used in laboratories performing calcium imaging (e.g., HuC/elavl3), *in vivo* nuclear staining dyes could provide a simple reference. Several cell-permeable DNA-binding dyes are available and are potentially applicable, but protocols need to be developed for live zebrafish larva. Alternatively, it could be explored whether autofluorescence, differential interference contrast, or IR-scanning gradient contrast imaging (Dodt et al., 1999; Wimmer et al., 2004) provide enough information to serve as a reference. An additional limitation is that the high spatial resolution of ViBE-Z currently comes at the cost of a large effort to record the high quality confocal data stacks. Therefore, algorithms suitable for lower quality data need to be developed in order to save microscope recording time. In parallel, the development and application of faster imaging techniques like selective plane illumination microscopy (SPIM; Huisken and Stainier, 2009) will also speed up and thus facilitate generation of *in vivo* data for integration into a reference atlas.

## Outlook

The integration of functional and anatomical data will advance our knowledge and facilitate interpretation of experimental results. Moreover, it should also improve the experimental design in the future. If one imagines a functional-anatomical atlas of the future, scientists could use the contained stereotactical information to guide their experiments in a hypothesis-driven fashion. For example, it could be attempted to only stimulate excitatory or inhibitory cells within a certain circuit module or to test different stimulation patterns that are chosen based on the expected connectivity in local circuits. In combination with advanced optical methods, the temporal and spatial photostimulation patterns could evolve to be as physiological as possible and test the combinatorial effects of multiplexed photostimulations in networks of neurons. These perturbation experiments, together with the projection patterns and expression markers in a zebrafish atlas of the future should open up new avenues of circuit analysis. It seems that zebrafish circuit neuroscience has a bright future in which advanced genetic, optogenetic, optical and computational methods will enable circuit analysis in three dimensions, at cellular resolution in the whole brain.

### Conflict of interest statement

The authors declare that the research was conducted in the absence of any commercial or financial relationships that could be construed as a potential *conflict of interest*.
